# Dissecting Colistin Resistance Mechanisms in Extensively Drug-Resistant Acinetobacter baumannii Clinical Isolates

**DOI:** 10.1128/mBio.01083-19

**Published:** 2019-07-16

**Authors:** Vincent Trebosc, Sarah Gartenmann, Marcus Tötzl, Valentina Lucchini, Birgit Schellhorn, Michel Pieren, Sergio Lociuro, Marc Gitzinger, Marcel Tigges, Dirk Bumann, Christian Kemmer

**Affiliations:** a BioVersys AG, Basel, Switzerland; b Biozentrum, University of Basel, Basel, Switzerland; McMaster University

**Keywords:** *Acinetobacter baumannii*, antibiotic resistance, colistin, *eptA*, ethanolamine transferase, *mcr-1*, *pmrA*

## Abstract

Nosocomial infections with Acinetobacter baumannii are a global problem in intensive care units with high mortality rates. Increasing resistance to first- and second-line antibiotics has forced the use of colistin as last-resort treatment, and increasing development of colistin resistance in A. baumannii has been reported. We evaluated the transcriptional regulator PmrA as potential drug target to restore colistin efficacy in A. baumannii. Deletion of *pmrA* restored colistin susceptibility in 10 of the 12 extensively drug-resistant A. baumannii clinical isolates studied, indicating the importance of PmrA in the drug resistance phenotype. However, two strains remained highly resistant, indicating that PmrA-mediated overexpression of the phosphoethanolamine (PetN) transferase PmrC is not the exclusive colistin resistance mechanism in A. baumannii. A detailed genetic characterization revealed a new colistin resistance mechanism mediated by genetic integration of the insertion element IS*AbaI* upstream of the PmrC homolog EptA (93% identity), leading to its overexpression. We found that *eptA* was ubiquitously present in clinical strains belonging to the international clone 2, and IS*AbaI* integration upstream of *eptA* was required to mediate the colistin-resistant phenotype. In addition, we found a duplicated IS*AbaI*-*eptA* cassette in one isolate, indicating that this colistin resistance determinant may be embedded in a mobile genetic element. Our data disprove PmrA as a drug target for adjuvant therapy but highlight the importance of PetN transferase-mediated colistin resistance in clinical strains. We suggest that direct targeting of the homologous PetN transferases PmrC/EptA may have the potential to overcome colistin resistance in A. baumannii.

## Abstract (precis)

The discovery of antibiotics revolutionized modern medicine and enabled us to cure previously deadly bacterial infections. However, a progressive increase in antibiotic resistance rates is a major and global threat for our health care system. Colistin represents one of our last-resort antibiotics that is still active against most Gram-negative bacterial pathogens, but increasing resistance is reported worldwide, in particular due to the plasmid-encoded protein MCR-1 present in pathogens such as Escherichia coli and Klebsiella pneumoniae. Here, we showed that colistin resistance in A. baumannii, a top-priority pathogen causing deadly nosocomial infections, is mediated through different avenues that result in increased activity of homologous phosphoethanolamine (PetN) transferases. Considering that MCR-1 is also a PetN transferase, our findings indicate that PetN transferases might be the Achilles heel of superbugs and that direct targeting of them may have the potential to preserve the activity of polymyxin antibiotics.

## INTRODUCTION

Antimicrobial resistance is a serious threat to global health systems, resulting in the loss of treatment options to fight a growing number of bacterial infections (1). Considering the paucity of newly developed antibiotics in the last decades, old antibiotics such as polymyxins have been increasingly used to treat infections caused by multidrug-resistant (MDR) Gram-negative pathogens (2–4). Nowadays, the polymyxin antibiotics polymyxin E (colistin) and polymyxin B represent the last resort for the treatment of serious Gram-negative infections, such as infections caused by carbapenem-resistant Enterobacteriaceae, MDR Pseudomonas aeruginosa, and MDR Acinetobacter baumannii (5, 6). Unfortunately, the increasing use of polymyxins to treat serious infections caused by these pathogens leads to a spread of resistance to these last-line drugs (7). There is a high unmet medical need for new drugs effective against Gram-negative bacteria to treat infections caused by these pathogens (8). Besides this, an alternative strategy resides in the recovery of colistin efficacy by blocking bacterial colistin resistance mechanisms. Antibiotic adjuvant therapies consist in the combination of a potent antibiotic with a nonantibiotic agent interfering with specific antibiotic resistance or virulence mechanisms. This strategy may provide a new tool to fight infections caused by drug-resistant pathogens by restoring or boosting the efficacy of an approved antibiotic (9).

Colistin resistance is conferred by lipopolysaccharide (LPS) modifications at the outer cell envelope. Reduction of the negative charge on LPS results in a reduced affinity of colistin to LPS (10). The two main LPS modifications conferring colistin resistance are the addition of 4-amino-4-deoxy-l-arabinose (AraN) and phosphoethanolamine (PetN) to the lipid A (11). The expression of LPS-modifying enzymes is regulated by the concerted action of several two-component systems (TCSs). In Enterobacteriaceae, PhoPQ and PmrAB TCSs regulate the expression of colistin resistance mechanisms, whereas in P. aeruginosa the PhoPQ, PmrAB, ParRS, ColRS, and CprRS TCSs seem to be involved (11). Plasmid-mediated colistin resistance has been recently reported in Enterobacteriaceae due to the PetN transferase MCR-1. The presence of MCR-1 on a plasmid leads to its rapid geographical and interspecies spread (12, 13). Nevertheless, *mcr-1* seems to be restricted to Enterobacteriaceae species and has never been detected in A. baumannii. In A. baumannii, colistin resistance is mediated by PetN addition to the lipid A, and this resistance mechanism is regulated by the PmrAB TCS. In contrast to other pathogens, the AraN lipid A modification pathway is not present in A. baumannii (11), rendering A. baumannii a suitable pathogen to develop an adjuvant therapy approach to rejuvenate colistin efficacy by blocking the PmrAB TCS.

Colistin resistance in A. baumannii clinical isolates is associated with alterations in the *pmrCAB* operon. The *pmrC* gene codes for a PetN transferase, and *pmrA* and *pmrB* code for the TCS (14). It has been shown that mutations in the PmrAB TCS induce the overexpression of *pmrC*, leading to the modification of lipid A with PetN and colistin resistance (14–18). Because PmrA is the transcriptional regulator that triggers PmrC overexpression, inhibition of PmrA with a small molecule may potentially block PmrC overexpression and therefore switch off colistin resistance in A. baumannii (19). This study was designed to evaluate the clinical relevance of PmrA as a drug target to restore colistin efficacy in A. baumannii. We demonstrate that in the absence of PmrA-mediated expression of PmrC, transposition of an insertion sequence (IS) element leads to overexpression of the alternative highly similar PetN transferase EptA, which also confers colistin resistance in A. baumannii clinical isolates. Our results show that in all studied clinical isolates, overexpression of at least one PetN transferase (PmrC or various EptA variants) was responsible for colistin resistance, indicating that PetN transferases may be a suitable drug target to overcome colistin resistance in A. baumannii.

## RESULTS

### PmrA is not essential for colistin resistance in A. baumannii clinical isolates.

We deleted *pmrA* from the genome of a panel of 12 colistin-resistant A. baumannii strains to evaluate the transcriptional regulator PmrA as a potential drug target to rejuvenate colistin efficacy in A. baumannii. The strains in the panel consisted of recently isolated colistin-resistant clinical strains collected from diverse geographical origins. They belong to three distinct and highly successful clonal lineages, the international clone 1 (ST1), international clone 2 (ST2), and ST25 clonal lineages (Table 1) (20–22). All strains were classified as extensively drug resistant according to the criteria of Magiorakos et al. (23). These data underscore the clinical relevance and the diversity of the strain panel. The colistin-susceptible A. baumannii ATCC 17978 strain was included as a reference strain. In all strains, *pmrA* was deleted by applying a previously described method that allows efficient scarless genome engineering even in extensively resistant A. baumannii clinical isolates (24).

**TABLE 1:** Characterization of the A. baumannii clinical isolate panel used in this study^*a*^

Strain designation	Strain isolation		MLST	MIC (μg/ml) of drug:							
	Country	Yr		GENT	MERO	CIP	TZP	CTX	SXT	SAM	TET
ATCC 17978	France	1951	77	*2*	*0.5*	*1*	*8/4*	16	**>8/152**	*4/2*	*2*
BV94	USA	2011	2	**>128**	**32**	**256**	**>256/4**	**>256**	**>8/152**	16/8	**32**
BV95	Colombia	2010	25	*1*	**64**	**128**	**256/4**	32	**>8/152**	16/8	**>256**
BV172	Israel	2012	2	**>128**	**64**	**32**	**256/4**	**>256**	**>8/152**	**64/32**	**>256**
BV173	Greece	2012	2	**>128**	**>64**	**128**	**>256/4**	**>256**	**>8/152**	**128/64**	**>256**
BV174	USA	2012	2	8	**64**	**256**	**256/4**	**256**	**>8/152**	**32/16**	**32**
BV175	Turkey	2012	2	**128**	**32**	**256**	**>256/4**	**256**	**>8/152**	**32/16**	**256**
BV185	Mexico	2013	2	**>128**	**>64**	**128**	**>256/4**	**>256**	**>8/152**	**64/32**	**256**
BV186	USA	2013	2	**16**	**64**	**256**	**>256/4**	**>256**	**>8/152**	**32/16**	8
BV187	USA	2013	2	**32**	**64**	**256**	**>256/4**	**>256**	**>8/152**	16/8	8
BV189	Spain	2013	2	**128**	**32**	**128**	**>256/4**	**256**	**>8/152**	**32/16**	**16**
BV190	Greece	2012	1	**>128**	**64**	**64**	**>256/4**	**>256**	**>8/152**	**64/32**	**256**
BV191	China	2013	2	**>128**	**>64**	**256**	**>256/4**	**>256**	**>8/152**	**128/64**	**>256**
ATCC 25922 (quality control)				1	<0.06	<0.25	4/4	<0.25	0.125/2.34	4/2	2

a Abbreviations: CIP, ciprofloxacin; CTX, cefotaxime; GENT, gentamicin; MERO, meropenem; MLST, multilocus sequence type; SAM, ampicillin-sulbactam; SXT, trimethoprim-sulfamethoxazole; TET, tetracycline; TZP, piperacillin-tazobactam. Classification of antibiotic resistance was done according to breakpoints published by the Clinical and Laboratory Standards Institute: susceptible (italics), intermediate (underlined), and resistant (bold) (34).

The colistin sensitivity of the parental clinical isolates and their corresponding *pmrA* knockout mutants (Δ*pmrA*) was determined by broth microdilution method. *pmrA* deletion reduced MICs 64- to 1,024-fold in 10 out of 12 initially colistin-resistant clinical isolates (83%), thus restoring susceptibility to colistin (MIC, ≤2 μg/ml) (Table 2). To our surprise, however, two strains (BV94 and BV189) retained colistin resistance even in the absence of *pmrA*.

**TABLE 2:** Effect of loss of PmrA on colistin susceptibility and PmrB mutations in the strain panel

Strain designation	Colistin MIC (μg/ml)^*a*^		PmrB mutations (amino acid substitutions)
	Wild type	Δ*pmrA*	
ATCC 17978	*0.25*	*0.25*	Reference
BV94	**64**	**32**	Wild type
BV95	**32**	*0.5*	L274W
BV172	**256**	*1*	Q43L and L267F
BV173	**128**	*1*	A138T and A226V
BV174	**64**	*1*	Q277R
BV175	**256**	*0.5*	L267W
BV185	**256**	*0.25*	P233S
BV186	**16**	*0.25*	Q277R
BV187	**16**	*0.25*	Q277R
BV189	**64**	**64**	Wild type
BV190	**256**	*0.5*	A138T and A226V
BV191	**256**	*0.25*	A138T and P233S

a Susceptibility breakpoint, ≤2 μg/ml. Susceptible, italics; resistant, bold.

**FIG 1:**
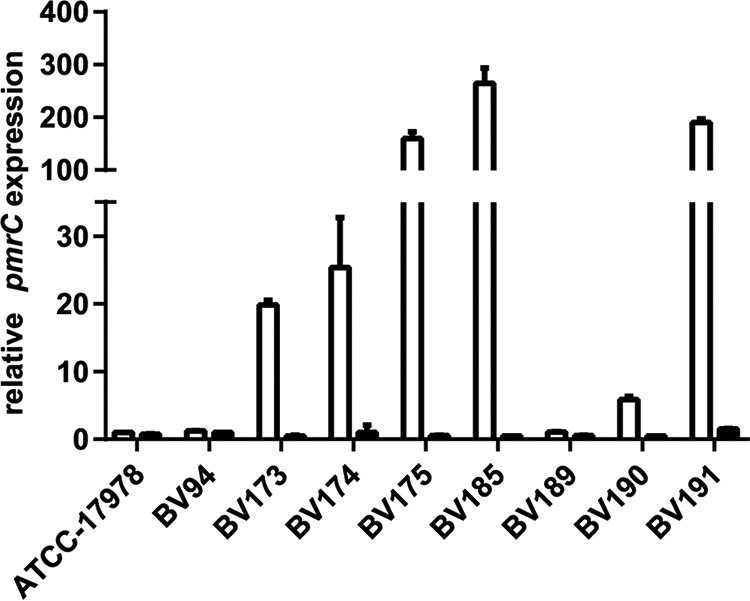
Quantification of *pmrC* expression levels in colistin-resistant A. baumannii clinical isolates and their Δ*pmrA* mutants. Expression levels of *pmrC* were quantified by qRT-PCR in colistin-resistant A. baumannii isolates (white bars) and their Δ*pmrA* mutants (black bars). The expression levels were normalized to the *pmrC* expression in the ATCC 17978 reference strain.

We investigated the differences between strains that became susceptible after *pmrA* deletion and those that remained resistant by analyzing the sequence variations of the PmrAB TCS in the strain panel. The PmrA and PmrB sequences of the A. baumannii AYE, ACICU, and NIPH 146 strains were used as references for ST1, ST2, and ST25 clonal lineages, respectively. Nonsynonymous mutations were found only in the PmrB sensor kinase (Table 2). Interestingly, the two strains with an unaltered PmrB sequence were those that remained colistin resistant after *pmrA* deletion (BV94 and BV189). Our data suggest that colistin resistance in these two strains is not conferred by PmrA-mediated PmrC overexpression. We confirmed this hypothesis by quantifying the expression of *pmrC* using quantitative reverse transcription-PCR (qRT-PCR) (Fig. 1). The control strain ATCC 17978 and the two refractory strains BV94 and BV189 showed only marginal levels of *pmrC* expression. In contrast, the 10 other strains showed *pmrC* overexpression, and this overexpression was abolished in the Δ*pmrA* mutant.

Taken together, colistin resistance in A. baumannii is predominantly conferred by mutations in the PmrB TCS sensor kinase that lead to overexpression of PmrC, as shown in 10 out of 12 clinical strains. However, some isolates (2 out of 12 strains in our panel) may use an alternative colistin resistance mechanism independent of PmrA-mediated PmrC overexpression to resist the antibacterial activity of colistin.

### EptA, a PmrC homolog, is present in the A. baumannii strains of international clone 2.

**FIG 2:**
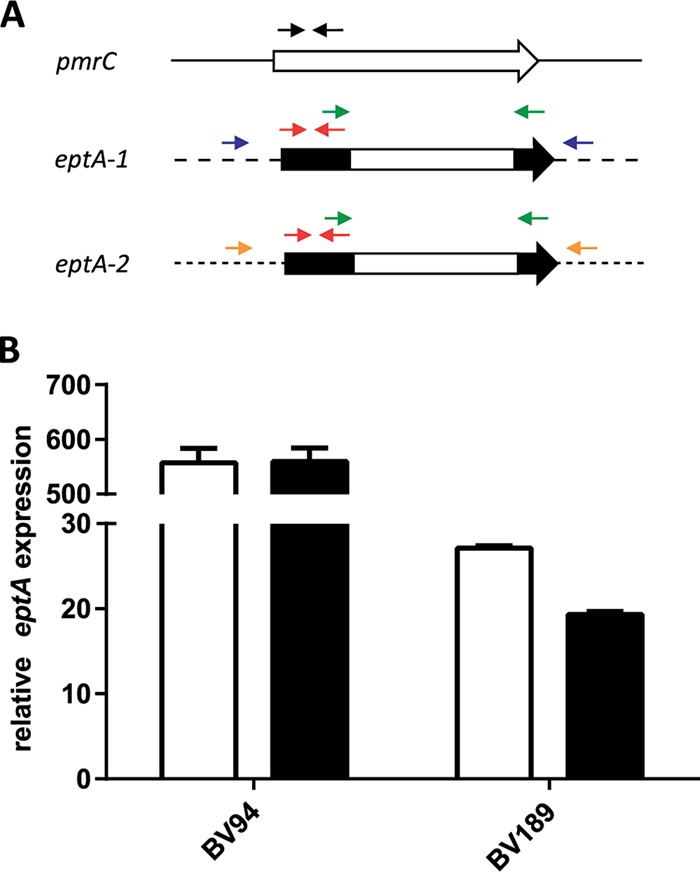
Discrimination and quantification of *pmrC* and *eptA*. (A) Schematic representation of differences in the *pmrC*, *eptA-1*, and *eptA-2* coding sequence. Primers marked by black (oVT162/oVT163) and red (oVT164/oVT165) arrows were used to detect *pmrC* and *eptA* in qRT-PCR experiments, respectively. The primers marked by green arrows (oVT152/oVT153) were used to genotype the *eptA* isoforms. Primers marked by blue (oVT198/oVT199) and orange (oVT201/oVT202) arrows were used to discriminate *eptA-1* and *eptA-2*, respectively. (B) Expression levels of *eptA* were quantified by qRT-PCR in colistin-resistant A. baumannii isolates BV94 and BV189 (white bars) and their Δ*pmrA* mutants (black bars). The expression levels were normalized to the *pmrC* expression in the ATCC 17978 reference strain.

Lesho et al. described the presence of the alternative PetN transferase EptA in A. baumannii (17). EptA and PmrC are homologous proteins with 93% amino acid identity, suggesting similar enzymatic activities. However, the role of EptA in A. baumannii colistin resistance is still unclear (17). To investigate the prevalence of *eptA* in A. baumannii, we took advantage of sequence differences between *pmrC* and *eptA* at the N- and C-terminal ends of the open reading frames and designed oligonucleotides (oVT152/oVT153) that can discriminate *eptA* from *pmrC* (Fig. 2A; see also Table S1 in the supplemental material). Using these *eptA*-specific primers, we detected *eptA* in all our international clone 2 strains but not in international clone 1 strains (Table 3 and Fig. S1). This finding was further confirmed by screening 12 additional isolates from the BioVersys strain collection (data not shown).

**TABLE 3:** Distribution of the *eptA* variants in the strain panel

Strain designation	MLST	*eptA* variant(s)
ATCC 17978	77	
BV94	2	*eptA-1*, IS*AbaI-eptA-2*, IS*AbaI-eptA-3*
BV95	25	
BV172	2	*eptA-1*
BV173	2	*eptA-1*
BV174	2	*eptA-1*, *eptA-2*
BV175	2	*eptA-1*
BV185	2	*eptA-1*
BV186	2	*eptA-1*, *eptA-2*
BV187	2	*eptA-1*, *eptA-2*
BV189	2	IS*AbaI-eptA-1*
BV190	1	
BV191	2	*eptA-1*

10.1128/mBio.01083-19.1 FIG S1 Agarose gel of *eptA-1* and *eptA-2* genotyping in the strain panel. The genotyping of *eptA-1* (upper gel) and *eptA-2* (lower gel) was performed by PCR using primers oVT198/oVT199 and oVT201/oVT202, respectively. Lane 1, 2-log ladder (New England Biolabs); lane 2, ATCC 17978; lane 3, BV94; lane 4, BV95; lane 5, BV172; lane 6, BV173; lane 7, BV174; lane 8, BV175; lane 9, BV185; lane 10, BV186; lane 11, BV187; lane 12, BV189; lane 13, BV190; lane 14, BV191; lane 15, 2-log ladder. Compared to the expected sizes of 1,862 bp and 1,827 bp, the PCR products for BV94 *eptA-2* and BV189 *eptA-1* genotyping, respectively, are approximately 1 kb larger, corresponding to the presence of IS*AbaI*. Download FIG S1, PDF file, 0.3 MB. Copyright © 2019 Trebosc et al. 2019 Trebosc et al. This content is distributed under the terms of the Creative Commons Attribution 4.0 International license.

10.1128/mBio.01083-19.3 TABLE S1 Oligonucleotides used in this study. Download Table S1, PDF file, 0.2 MB. Copyright © 2019 Trebosc et al. 2019 Trebosc et al. This content is distributed under the terms of the Creative Commons Attribution 4.0 International license.

### The integrated insertion element IS*AbaI* causes *eptA* overexpression in BV94 and BV189.

**FIG 3:**
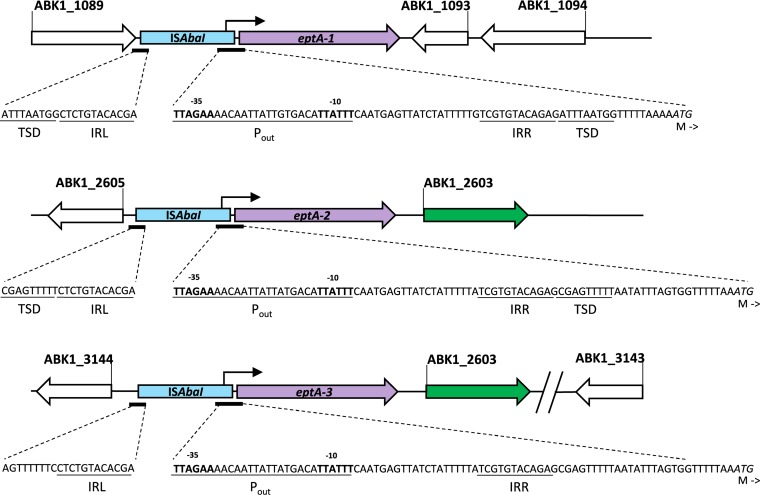
Representation of the different IS*AbaI-eptA* genomic regions present in BV94 and BV189. The nucleic acid sequence of the IS*AbaI* inverted repeats right and left (IRR and IRL, respectively) and P_out_ promoter are shown until the *eptA* start codon. The 9-bp target site duplications (TSD) up- and downstream of IS*AbaI* are not present for *eptA-3*, which is consistent with an IS*AbaI-eptA-2* duplication. The junction between ABK1_3144 and IS*AbaI-eptA-3* has been sequenced, while the sequence downstream of ABK1_2603 could not be resolved. The ABK1 gene annotation is shown according to the genomic sequence of A. baumannii strain 1656-2 (GenBank accession number NC_017162).

Two isoforms of *eptA*, *eptA-1* and *eptA-2* (GenBank accession numbers KC700024 and KC700023, respectively) have been described at different locations in the genome of various A. baumannii strains (17). Taking advantage of the different flanking regions, we designed primers able to discriminate *eptA-1* from *eptA-2* (oVT198/oVT199 and oVT201/oVT202, respectively) (Fig. 2A and Table S1). By genotyping the strain panel, we demonstrated that all strains that belong to the international clone 2 contained *eptA-1* and four of them contained an additional copy of *eptA-2* (Table 3 and Fig. S1). Interestingly, the PCR products obtained for *eptA-2* in BV94 and *eptA-1* in BV189 were approximately 1 kb larger than the expected fragment size. Sequencing of the PCR products identified the insertion element IS*AbaI* upstream of *eptA-2* and *eptA-1* in BV94 and BV189, respectively. The IS*AbaI* orientation enabled its strong promoter (*P_out_*) to drive *eptA* overexpression as previously described for other antibiotic resistance determinants (Fig. 3) (25, 26).

Using probes specific for *eptA* or *pmrC*, we quantified their respective expression levels in our strains. *eptA* was 550- and 25-fold higher expressed in BV94 and BV189, respectively, than the homologous isoform *pmrC* in the control strain ATCC 17978 (which does not contain *eptA*) (Fig. 2B). This *eptA* overexpression was not altered in the Δ*pmrA* mutant strains, suggesting that *eptA* expression in both strains is independent of the PmrAB TCS. These data suggest that an IS*AbaI*-driven *eptA* overexpression may represent an alternative and PmrAB-independent colistin resistance mechanism in A. baumannii clinical strains.

### IS*AbaI*-driven *eptA* overexpression confers colistin resistance in A. baumannii clinical isolates.

To validate the hypothesis that IS*AbaI*-driven *eptA* overexpression confers colistin resistance in A. baumannii clinical isolates, we deleted *eptA-1* in the clinical isolates BV189 and BV94 and determined MIC values. Indeed, BV189 (which carries IS*AbaI* upstream of *eptA-1*) became colistin susceptible upon *eptA-1* deletion, indicating an essential role of IS*AbaI*-*eptA-1* in conferring colistin resistance in this strain (Table 4). In contrast, BV94, carrying both *eptA-1* and *eptA-2* isoforms but carrying an IS*AbaI* insertion only upstream of *eptA-2*, remained colistin resistant after deletion of *eptA-1*, suggesting the key role of IS*AbaI* insertion for colistin resistance in A. baumannii. To further confirm the importance of IS*AbaI*, we constructed the double mutant BV94_Δ_*_eptA-1/_*_Δ_*_eptA-2_* and evaluated its colistin susceptibility. A 4-fold MIC reduction was observed in the BV94_Δ_*_eptA-1/_*_Δ_*_eptA-2_* mutant compared to BV94 and BV94_Δ_*_eptA-1_*, indicating that *eptA-2* with an upstream IS*AbaI* is involved in the colistin resistance mechanism of BV94. However, we were surprised to see that BV94_Δ_*_eptA-1/_*_Δ_*_eptA-2_* remained resistant to colistin with a MIC of 16 μg/ml, indicating that there must be yet another colistin resistance mechanism present in this isolate.

**TABLE 4:** Recovery of colistin susceptibility after deletion of different *eptA* isoforms

Strain	Colistin MIC (μg/ml)^*a*^			
	Wild type	Δ*eptA-1*	Δ*eptA-1/*Δ*eptA-2*	Δ*eptA-1/*Δ*eptA-2/*Δ*eptA-3*
BV189	**128**	*0.5*		
BV94	**64**	**64**	**16**	*1*

a Susceptibility breakpoint, 2 μg/ml. Susceptible, italics; resistant, bold.

### Three different *eptA* variants can confer colistin resistance in A. baumannii.

We genotyped the BV94_Δ_*_eptA-1/_*_Δ_*_eptA-2_* double mutant and confirmed the successful deletion of *eptA-1* and *eptA-2*. However, we detected the presence of at least one additional *eptA* copy (*eptA-3*) in the double mutant (Fig. S2). We performed a fusion primer and nested integrated PCR experiment (FPNI-PCR) to amplify the genomic flanking regions of the additional *eptA-3* variant (27). Sequencing revealed the IS*AbaI* insertion element and the gene ABK1_2603 present upstream and downstream of *eptA-3*, respectively (Fig. 3). This *eptA-3* gene context in BV94 was identical to the *eptA-2* gene context present in the A. baumannii strain 1656-2 (GenBank accession number NC_017162). However, further upstream there were marked differences. IS*AbaI-eptA-3* in BV94 was adjacent to the gene ABK1_3144, while IS*AbaI*-*eptA-2* in 1656-2 was adjacent to a different gene (Fig. 3). We could not determine the downstream flanking region of IS*AbaI*-*eptA3* ABK1_2603 in multiple attempts. Nevertheless, the 9-bp target site duplications (TSD) created by IS*AbaI* transposition could not be identified directly outside the IS*AbaI* upstream *eptA-3* (25). In contrast, TSD were present next to the right and left inverted repeats of the IS*AbaI* upstream *eptA-1* and *eptA-2*, which is consistent with a single transposition event. These observations indicate that IS*AbaI* did not insert upstream *eptA-3* in a single transposition event, and therefore, IS*AbaI-eptA-3* in BV94 might be a result of an IS*AbaI-eptA-2* cassette gene duplication, implying that the IS*AbaI-eptA* colistin resistance determinant is contained in a mobile genetic element.

10.1128/mBio.01083-19.2 FIG S2 Agarose gel of *eptA* genotyping in the clinical isolate BV94 and its *eptA* knockout mutants. The genotyping of *eptA* isoforms was performed by PCR using primers oVT152/oVT153. Lane 1, 2-log ladder (New England Biolabs); lane 2, BV94; lane 3, BV94_Δ_*_eptA-1_*; lane 4, BV94_Δ_*_eptA-1_*_/Δ_*_eptA-2_*; lane 5, BV94_Δ_*_eptA-1_*_/Δ_*_eptA-2_*_/Δ_*_eptA-3_*. The triple mutant BV94_Δ_*_eptA-1_*_/Δ_*_eptA-2_*_/Δ_*_eptA-3_* did not carry any other *eptA* isoform. Download FIG S2, PDF file, 0.3 MB. Copyright © 2019 Trebosc et al. 2019 Trebosc et al. This content is distributed under the terms of the Creative Commons Attribution 4.0 International license.

We finally deleted the DNA fragment between ABK1_3144 and ABK1_3143 containing *eptA-3* to confirm that IS*AbaI-eptA-3* was responsible for the high residual colistin resistance in BV94_Δ_*_eptA-1/_*_Δ_*_eptA-_*_2_. In addition, we performed PCR-based *eptA* genotyping on the resulting triple mutant BV94_Δ_*_eptA-1/_*_Δ_*_eptA-2/_*_Δ_*_eptA-3_* to exclude the presence of yet another *eptA* copy (Fig. S2). The loss of all 3 *eptA* isoforms in BV94_Δ_*_eptA-1/_*_Δ_*_eptA-2/_*_Δ_*_eptA-3_* rendered this triple mutant susceptible to colistin, indicating that colistin resistance in BV94 was entirely conferred by the overexpression of EptA-isoforms (Table 4).

### Targeting PetN transferases may overcome colistin resistance in A. baumannii.

**FIG 4:**
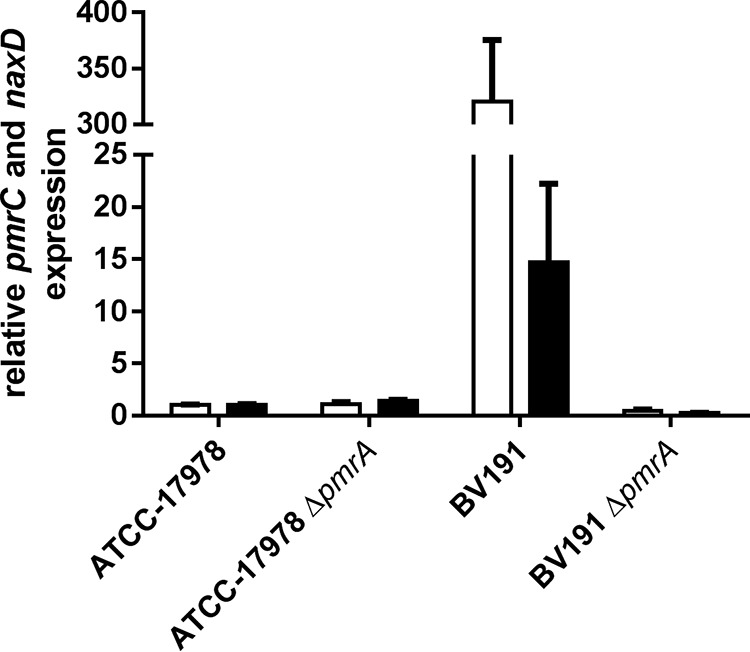
Quantification of *pmrC* and *naxD* expression levels in A. baumannii BV191 and its Δ*pmrA* mutants. The expression of *pmrC* (white bars) and *naxD* (black bars) was quantified by qRT-PCR and normalized to the gene expression level in the reference strain ATCC 17978.

We have shown that colistin resistance was mediated in 10 out of 12 analyzed clinical strains by PmrA-mediated overexpression of PmrC. In the remaining two strains, IS*AbaI*-driven EptA expression conferred colistin resistance. Taken together, in all tested clinical isolates colistin resistance was mediated by the overexpression of PetN transferases, suggesting that inhibition of these homologous enzymes with small molecules may have the potential to overcome colistin resistance in A. baumannii. Chin and colleagues recently suggested that the acetyl-galactosamine (GalNAc) deacetylase NaxD plays a role in colistin resistance in A. baumannii (28). In this report, the expression of NaxD, which was regulated by the PmrAB TCS, mediated galactosamine (GalN) addition to lipid A, conferring colistin resistance in A. baumannii. We performed additional experiments to exclude the possibility that the colistin resensitization observed in our clinical isolates after deletion of *pmrA* was based on a modulation of *naxD* expression and not *pmrC* expression. We first confirmed the PmrAB-controlled *naxD* expression based on qRT-PCR data for BV191 and BV191_Δ_*_pmrA_*. BV191 has a mutated PmrB that likely triggers PmrA-mediated *pmrC* overexpression (Table 2). Similarly, *naxD* expression was 15-fold higher in the colistin-resistant strain BV191 than the susceptible strain ATCC 17978 (Fig. 4). In BV191_Δ_*_pmrA_*, lacking the response regulator PmrA, *pmrC* and *naxD* overexpression was abolished, confirming that both genes were regulated by the PmrAB TCS. Notably, *pmrC* overexpression was 20-fold higher than *naxD* overexpression, suggesting a minor contribution of NaxD compared to PmrC in colistin resistance. To confirm the major role of PmrC in PmrA-mediated colistin resistance and to exclude that another PmrA-regulated gene, such as *naxD*, is involved in colistin resistance, we directly deleted the effector *pmrC* from the genome of BV191. The loss of PmrC rendered BV191 susceptible to colistin (MIC of 0.5 μg/ml) and resulted in a similar phenotype as in BV191_Δ_*_pmrA_* (Table 2). In contrast, *naxD* was still 15-fold overexpressed in BV191_Δ_*_pmrC_* (Fig. 4). This result suggests that overexpression of *naxD* is not sufficient to confer colistin resistance in BV191 and indicates that PmrC is the main effector of PmrA-mediated colistin-resistant A. baumannii strains, such as BV191.

## DISCUSSION

Bacteria have evolved multiple ways to escape the hazardous action of antibiotics. In nosocomial infections, the individual strain history of antibiotic exposures during patient treatment may result in the development and accumulation of different resistance mechanisms in different strains of the same species. Therefore, it is important to study resistance mechanisms on multiple strains. Moreover, it is crucial to study these mechanisms on strains that developed resistance during patient treatment due to the discrepancy that may be observed between *in vitro*- and *in vivo*-developed mechanisms. For instance, A. baumannii polymyxin resistance is commonly mediated by LPS loss when A. baumannii is exposed to the drug *in vitro*, but this mechanism is not viable *in vivo* due to the strong fitness cost that it engenders (16, 29).

In this study, we dissected the mechanisms conferring colistin resistance in 12 clinically relevant A. baumannii strains. To our knowledge, this is the first time that colistin resistance is genetically characterized in a panel of A. baumannii clinical strains that developed resistance during patient treatment and not strains that artificially acquired resistance by *in vitro* selection/passaging. This gap in knowledge originates from the difficulties in manipulating the genome of A. baumannii colistin-resistant clinical strains. Indeed, as exemplified in our strain panel, such strains are generally resistant to all other antibiotics because colistin is used as a last option in the treatment of A. baumannii infections, only when other antibiotics fail. To break the barrier of antibiotic resistance in these strains, we applied a genome editing method based on a nonantibiotic resistance marker, which is efficient regardless of the resistance profile of the strain (24).

**FIG 5:**
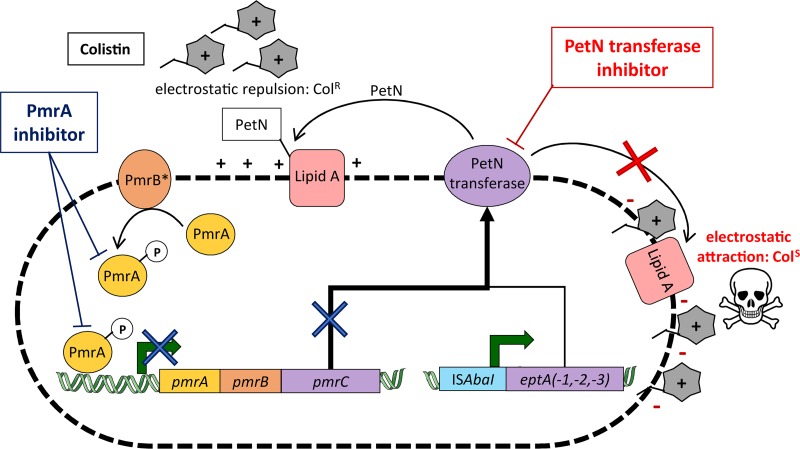
Schematic representation of A. baumannii colistin resistance mechanisms. The two pathways leading to phosphoethanolamine (PetN) transferase overexpression and colistin resistance are represented. The major A. baumannii PetN transferase overexpression pathway results from *pmrC* expression, which is activated by the transcriptional regulator PmrA previously phosphorylated (activated) by a mutated variant of the sensor kinase PmrB (PmrB*). Alternatively, A. baumannii PetN transferase overexpression can result from the integration of the IS*AbaI* insertion element upstream of an *eptA* isoform. PetN transferase enzymes decorate the outer membrane lipid A with PetN, thereby lowering the negative charge and preventing colistin binding. Potential PmrA inhibitors would only block the *pmrC* pathway (dark blue cross), while PetN transferase inhibitors would block lipid A modification (red cross) and restore colistin efficacy against A. baumannii.

We demonstrated two different ways to overexpress PetN transferases that cause colistin resistance in A. baumannii clinical isolates (Fig. 5). The predominant colistin resistance mechanism found in 83% of the studied clinical isolates was mediated by *pmrC* overexpression. The overexpression of *pmrC* in these strains was entirely caused by mutations in the sensor kinase PmrB, although previous studies also found mutations in the response regulator PmrA (14, 15, 17). We found 7 different PmrB variants among the 10 PmrC-mediated colistin-resistant strains, indicating the diversity of mutations that lead to PmrC overexpression. Except fo r the A226V and P233S mutations, the identified PmrB mutations were not yet reported in A. baumannii (11, 15, 16).

Interestingly, we found two clinical isolates in which colistin resistance was conferred by a genomic insertion of IS*AbaI*, resulting in a strong overexpression of the *pmrC* homolog *eptA*. *eptA-1* and *eptA-2* genes have been previously identified in A. baumannii; however, their distribution, expression regulation, and role in colistin resistance were not assessed (17). Our study revealed that A. baumannii strains of the international clone 2, which represent the most problematic strains in hospitals, carry at least one *eptA* variant. In contrast, international clone 1 strains did not carry *eptA*. Our data further show that *eptA* expression is not regulated by the PmrAB TCS, but instead, integration of IS*AbaI* upstream of any *eptA* isoform is required to confer the resistance phenotype, presumably by IS*AbaI*-driven *eptA* overexpression. Consequently, detection of an *eptA* gene alone is not sufficient to classify A. baumannii strains as colistin resistant.

The analysis of PmrA as a potential drug target confirmed the importance of this protein in mediating colistin resistance in A. baumannii. However, the high prevalence of *eptA* and the ability of IS*AbaI* to integrate upstream of *eptA* and drive its expression independently of the PmrAB TCS disproved PmrA as a direct drug target for resensitization of A. baumannii to colistin (Fig. 5). An adjuvant therapy consisting of a PmrA inhibitor in combination with colistin would most likely select for IS*AbaI*-driven EptA-overexpressing colistin-resistant strains. As demonstrated by the two clinical isolates BV94 and BV189, such strains are already present in hospitals. One of the strains also contained a duplicated IS*AbaI-eptA* cassette, suggesting that this functional cassette mediating colistin resistance was present on a mobile element. The presence of a mobile colistin-resistance-mediating cassette increases the probability of intra- and interspecies transfer of the resistance pathways by the integration into plasmids. This phenomenon was recently illustrated with plasmid-carried PetN transferase *mcr-1*, which was initially found in China but rapidly has spread globally and in different species (12, 13). Nevertheless, *mcr-1* seems to be limited to Enterobacteriaceae species and has never yet been detected in A. baumannii.

One of the major colistin resistance pathways in Enterobacteriaceae and P. aeruginosa is the addition of AraN to lipid A (11). Although we describe here two different ways to overexpress PetN transferases, our results suggest that colistin resistance in clinical A. baumannii isolates is exclusively conferred by PetN addition to lipid A. A recent study suggested that a GalN-based modification of lipid A may be involved in colistin resistance in A. baumannii (28). In contrast, our results suggest that alteration of the lipid A structure by addition of PetN plays the major role in colistin resistance in A. baumannii. It has also been described that loss of LPS may confer colistin resistance in A. baumannii (30). However, most of the LPS-deficient colistin-resistant mutants were obtained *in vitro* after colistin evolution, and it has been shown that these mutants are hypersusceptible to other antibiotic classes and are avirulent (16, 29). Emergence of LPS-deficient colistin-resistant mutants in patients is therefore unlikely.

In conclusion, the overexpression of homologous PetN transferases caused colistin resistance in all studied clinical isolates, but in some cases this occurred independently of PmrAB. The crystal structure of Neisseria meningitidis PetN transferase has been recently reported, and this enzyme has been proposed as a drug target for antivirulence and antiresistance drug development to treat Neisseria gonorrhoeae and N. meningitidis infections (31, 32). Our data suggest that a direct inhibitor of homologous PetN transferases PmrC and EptA may have the potential to overcome colistin resistance in A. baumannii clinical strains (Fig. 5).

## MATERIALS AND METHODS

### Bacterial strains, MIC, MLST, and oligonucleotides.

The A. baumannii reference strain ATCC 17978 and 12 extensively drug-resistant A. baumannii clinical isolates from the BioVersys proprietary strain collection were used in this study. The microdilution method was used to determine MICs according to the CLSI guidelines (33). Multiple locus sequence type (MLST) was determined according to the Pasteur scheme using specific primers (source: http://pubmlst.org/abaumannii/) (20). Oligonucleotides used in this study are listed in Table S1 in the supplemental material.

### Genomic deletions of *pmrA*, *eptA-1*, *eptA-2*, *eptA-3*, and *pmrC* in A. baumannii clinical isolates.

Scarless deletions of *pmrA*, *pmrC*, and the *eptA* isoforms were performed using a two-step recombination method previously described (24).

DNA fragments corresponding to 700-bp up- and downstream genomic regions of the genes to be deleted were amplified by PCR and cloned in the multiple cloning site of the knockout platform pVT77. Oligonucleotides oVT49/oVT50 and oVT51/oVT52 were used to amplify the up- and downstream regions, respectively, of *pmrA.* The resulting DNA fragments were ligated and introduced into pVT77 previously digested by EcoRI and BamHI. Similarly, oligonucleotides oVT235/oVT236 and oVT237/oVT238 were used to amplify the flanking regions of *eptA-1*, and oligonucleotides oVT305/oVT306 and oVT307/oVT242 were used to amplify the flanking regions of *eptA-2.* The resulting DNA fragments for *eptA-1* and *eptA-2* were introduced into pVT77 previously digested by XhoI and XbaI using NEBuilder HiFi DNA assembly (New England Biolabs). For *eptA-3* deletion, the genomic regions flanking the duplicated cassette were amplified using oVT390/oVT391 and oVT392/oVT393. The resulting DNA fragments were cloned into pVT77 previously digested with EcoRI and XbaI using NEBuilder HiFi DNA assembly. Last, the flanking regions of *pmrC* were amplified using oVT324/oVT325 and oVT326/oVT327, and the resulting DNA fragments were cloned into pVT77 previously digested with KpnI and PstI using NEBuilder HiFi DNA assembly.

The cloned knockout plasmids were transformed in E. coli conjugative strain MFD*pir* to proceed with the construction of markerless deletion in A. baumannii, as previously described (24). Briefly, after conjugation, genomic plasmid integration was selected on LB agar plates containing 100 μg/ml sodium tellurite. Clones were screened for up- or downstream integration by PCR using primer oVT8, which anneals on the plasmid, and oVT91, oVT243, oVT311, or oVT328, which anneals upstream of *pmrA*, *eptA-1*, *eptA-2*, or *pmrC*, respectively. For *eptA-3*, clones were screened using primers oVT8/oVT396 and oVT174/oVT397 for up- and downstream integration, respectively. Clones containing up- and downstream plasmid integrations were transferred on LB agar plates containing 1 mM isopropyl-β-d-1-thiogalactopyranoside and 200 μg/ml 3′-azido-3′-deoxythymidine to select for plasmid removal from the genome. Clones were screened for gene deletion and plasmid removal by PCR using primers oVT91/oVT92, oVT243/oVT244, oVT246/oVT311, oVT396/oVT397, and oVT328/oVT14 for *pmrA*, *eptA-1*, *eptA-2*, *eptA-3*, and *pmrC*, respectively. The genomic gene deletions were finally confirmed by DNA sequencing (Microsynth AG, Balgach, Switzerland).

### Genotyping of *pmrA*, *pmrB*, and *eptA*.

A genomic DNA sequence including *pmrA* and *pmrB* was PCR amplified from all the strains of the panel using oVT91 and oCK292, and the PCR products were sent for sequencing (Microsynth AG, Balgach, Switzerland). The genotyping of *eptA* isoforms was performed by PCR using *eptA*-specific primers oVT152 and oVT153, which anneal on all *eptA* isoforms but not on *pmrC* (Fig. 2A). PCR using primers oVT198/oVT199 and oVT201/oVT202, which anneal on the flanking sides of *eptA-1* and *eptA-2*, respectively, were used to discriminate between *eptA* isoforms (Fig. 2A).

### qRT-PCR.

Quantitative reverse transcription-PCR was performed as previously described (24). The specific expression of the PetN transferases encoded by *pmrC* and *eptA* was evaluated using oVT162/oVT163 and oVT164/oVT165 primers, respectively (Fig. 2A). The expression of *naxD* was evaluated using oVT314/oVT315 primers. Expression levels were normalized to that of the housekeeping gene *rpoD* using the comparative threshold cycle (ΔΔ*C_T_*) method. The expression of *rpoD* was evaluated using rpoD-qRT-F/rpoD-qRT-R primers.

### FPNI-PCR.

Fusion primer and nested integrated PCR was performed as previously described (27). This method relies on a three-step PCR using arbitrary degenerated oligonucleotides fused to known adaptors and three sequence-specific oligonucleotides, which consist in our case of *eptA*-specific oligonucleotides. FPNI-PCR experiments were performed on the BV94_Δ_*_eptA-1/_*_Δ_*_eptA-2_* mutant with two sets of three *eptA*-specific oligonucleotides, oligonucleotides oVT343, oVT344, and oVT345 and oligonucleotides oVT340, oVT341, and oVT342, to identify the sequence up- and downstream of the new *eptA* copy, respectively. The degenerated primers and the known adaptor primers were directly taken from the previously described method (27). Briefly, the first round of PCRs was performed using the degenerated primers and oVT343 for upstream identification and oVT340 for downstream identification. The second round of PCRs was performed with the first adaptor primer FSP1 and oVT344 for upstream identification and oVT341 for downstream identification. The last round of PCRs was performed with the second adaptor primer FSP2 and oVT345 for upstream identification and oVT342 for downstream identification. The brightest and most distinct PCR products obtained were sent for sequencing (Microsynth AG, Balgach, Switzerland).
