# Supplementary material for: Dissecting Colistin Resistance Mechanisms in Extensively Drug-Resistant Acinetobacter baumannii Clinical Isolates
Source: mBio. 2019 Jul 16;10(4):e01083-19. doi: 10.1128/mBio.01083-19 (PMC6635527; doi:10.1128/mBio.01083-19)
Supplement: FIG S1 [file mBio.01083-19-sf001.pdf]

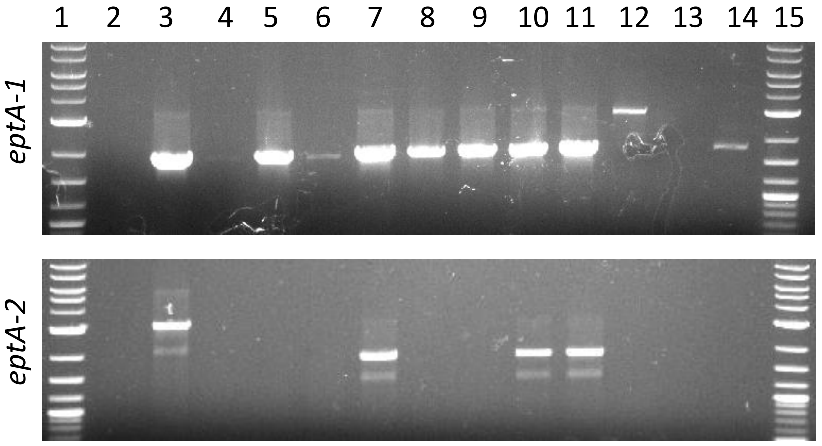

Fig S1: Agarose gel of *eptA-1* and *eptA-2* genotyping in the strain panel.

The genotyping of *eptA-1* (upper gel) and *eptA-2* (lower gel) was performed by PCR using primers oVT198/oVT199 and oVT201/oVT202, respectively. Lane 1: 2-log ladder (New England Biolabs), lane 2: ATCC-17978, lane 3: BV94, lane 4: BV95, lane 5: BV172, lane 6: BV173, lane 7: BV174, lane 8: BV175, lane 9: BV185, lane 10: BV186, lane 11: BV187, lane 12: BV189, lane 13: BV190, lane 14: BV191 and lane 15: 2-log ladder. Compared to the expected size of 1,862-bp and 1,827-bp, the PCR products for BV94 *eptA-2* and BV189 *eptA-1* genotyping are approximately 1 kilobase larger corresponding to the presence of IS*AbaI*.
