# Supplementary material for: Dissecting Colistin Resistance Mechanisms in Extensively Drug-Resistant Acinetobacter baumannii Clinical Isolates
Source: mBio. 2019 Jul 16;10(4):e01083-19. doi: 10.1128/mBio.01083-19 (PMC6635527; doi:10.1128/mBio.01083-19)
Supplement: TABLE S1 [file mBio.01083-19-st001.pdf]

Table S1: Oligonucleotides used in this study

Oligo name	Sequence (5'-3')
oVT8	GTTTTCCCAGTCACGACGC
oVT14	TGAGGTGCCCCAAATCAGTC
oVT49	GGAATTCCGGTTACCAAGTGACTTGG
oVT50	CGAAATTTTAAATCCCCCGGGGGAGTTCTAGGCTCGCTTTAGTTTAC
oVT51	CTCCCCCGGGGGATTTAAAATTTCGGGACTTC
oVT52	CGGATCCGTCGTAATTCATGAGCAGC
oVT91	CGGGTATGCCACGTGTAGAT
oVT92	CAAATGCTGAATACGCGCCA
oVT152	AGTTCTTTTCTTAGGGGCGACA
oVT153	ACATTGGCTCAACATATCAAGTTTGT
oVT162	CCATTTGGCTAGGTGCAATTT
oVT163	ACCGCATAATAGGTAGCAACA
oVT164	TGCTTTTATCTATCTGGCTAGGTT
oVT165	TGTCGCCCCTAAGAAAAGAACT
oVT174	ACCCAGCATGATATTCCGGA
oVT198	CTCAAGGAGCCATCGTCATAT
oVT199	GGGTTACGACTGCCCTCTTA
oVT201	AGTTTTACTAGCTAAGATTTCGAG
oVT202	TGATGCATAGCTGACGAAG
oVT235	GGAGAATTGAGGCCTCTCGAGCATTCAGGTGTTGCTGTG
oVT236	CCCTCTTACCCGGGATATGACGATGGCTCCTTG
oVT237	TCGTCATATCCCGGGTAAGAGGGCAGTCGTAACC
oVT238	AGCTTCCTGCAGGCTCTAGAAATGGGGGCTTCTGG
oVT242	AGCTTCCTGCAGGCTCTAGACGCCCTTTGGTCTCCTAAC
oVT243	TATGGCACAAGAAGAAGGCG
oVT244	ACAGCTTGGCCATAGGAAGC
oVT246	GCTTTTGTGAAGTTGGGGCC
oVT305	GGAGAATTGAGGCCTCTCGAGACGATTGCGAGCATCAGG
oVT306	ACGAAGGTCCCGGGCGAAACATCGGTCTGCAC
oVT307	GATGTTTCGCCCGGGACCTTCGTCAGCTATGCATC
oVT311	ATGTAAACCACTGCTCACCG
oVT314	TGGCAAAGGTGTGCTATTGC
oVT315	ATTTGGCAGCAGCAGTTTCC
oVT324	AATTCGAGCTCGGTACCAGAGGTTCTGCTTCAGCTC
oVT325	CTCGCTTTACCCGGGAAACAGGGAAATCTGTTTATTTC
oVT326	CCCTGTTTCCCGGGTAAAGCGAGCCTAGAACATG
oVT327	CGCAAGCTTCCTGCAGATTGAGGTGCCCCAAATC
oVT328	TCGACAAACATTGGTGGTGG
oVT340	AGCGGTTTCTGTGCCATGTA
oVT341	CCGCGTTTGATTGTTCTGCA
oVT342	TACAGGGCTGTTCGCAAACT
oVT343	GCAGGTCCATGACTACCCAC
oVT344	TGCAGAACAATCAAACGCGG
oVT345	CCGACCACTGCAAATGAAGC
oVT390	AGAATTGAGGCCTCTCGAGGAATTCATATGTGTTGTGGTCTGGG
oVT391	TGGGATTTTTCCCGGGGACCCACAAAATCTCTTTTC
oVT392	TTTGTGGGTCCCCGGGAAAAATCCCACCGAAGTG
oVT393	CCGCAAGCTTCCTGCAGGCTCTAGAAAACGTGCTCAAACTGC
oVT396	TAAATCCTGGCACTTGGTGG
oVT397	CCGGTGGTATGGAGTCGATG
oCK292	GGACAGGCTGGGTCGTTTG
rpoD-qRT-F	GAGTCTAATGGCGGTGGTTC
rpoD-qRT-R	ATTGCTTCATCTGCTGGTTG
