# Supplementary material for: Dissecting Colistin Resistance Mechanisms in Extensively Drug-Resistant Acinetobacter baumannii Clinical Isolates
Source: mBio. 2019 Jul 16;10(4):e01083-19. doi: 10.1128/mBio.01083-19 (PMC6635527; doi:10.1128/mBio.01083-19)
Supplement: FIG S2 [file mBio.01083-19-sf002.pdf]

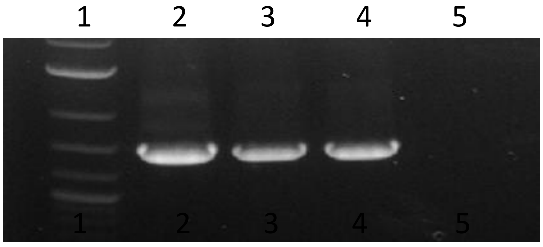

Fig S2: Gel agarose of *eptA* genotyping in the clinical isolate BV94 and its *eptA* knockout mutants. The genotyping of *eptA* isoforms was performed by PCR using primers oVT152/oVT153. Lane 1: 2-log ladder (New England Biolabs), lane 2: BV94, lane 3: BV94$_{\Delta eptA\text{-}1}$, lane 4: BV94$_{\Delta eptA\text{-}1/\Delta eptA\text{-}2}$, lane 5: BV94$_{\Delta eptA\text{-}1/\Delta eptA\text{-}2/\Delta eptA\text{-}3}$. The triple mutant BV94$_{\Delta eptA\text{-}1/\Delta eptA\text{-}2/\Delta eptA\text{-}3}$ did not encode any other *eptA* isoform.
